# Environment‐susceptible genetic loci for imaging‐based preclinical markers of ADRD, and the influence of modifiable risk factors

**DOI:** 10.1002/alz70855_103860

**Published:** 2025-12-24

**Authors:** Sathyaseelan Chakkarai, Mohsen Sharifi Tabar, Xueqiu Jian, Claudia L Satizabal, Francesco Paolo Casale, Juan B Gutiérrez, Mohamad Habes, John Blangero, Sudha Seshadri, Muralidharan Sargurupremraj

**Affiliations:** ^1^ University of Texas Health Science Center at San Antonio, San Antonio, TX, USA; ^2^ The University of Texas Health Science Center at San Antonio, San Antonio, TX, USA; ^3^ University of Texas Health San Antonio, San Antonio, TX, USA; ^4^ Helmholtz Zentrum München ‐ German Research Center for Environmental Health, Neuherberg, Bavaria, Germany; ^5^ Technical University of Munich, Munich, Bavaria, Germany; ^6^ The University of Texas at San Antonio, San Antonio, TX, USA; ^7^ Division of Human Genetics, University of Texas Rio Grande Valley, Brownsville, TX, USA; ^8^ Department of Neurology, Boston University School of Medicine, Boston, MA, USA

## Abstract

**Background:**

Epidemiological studies highlight the relation between environmental exposures and the risk of Alzheimer's disease (AD) and related disorders (ADRD). However, due to the prolonged preclinical phase of ADRD, the underlying molecular mechanisms disrupted by environmental factors and their impact on brain structure remain poorly understood. Additionally, investigating gene‐environment interactions has been challenging, primarily due to difficulties in accurately defining environmental variables and the substantial inter‐study heterogeneity.

**Method:**

Since gene‐environment interaction effects influence phenotypic variability across genotypes, we leveraged deviations in phenotypic variance to identify variance quantitative trait loci (vQTLs) with heightened environmental sensitivity. Utilizing UK Biobank data (*N* = 45,275) with comprehensive genomic, brain imaging, and risk factor profiles, we mapped vQTLs for key MRI markers: hippocampal volume (HV) for atrophy, white matter hyperintensity (WMH) for vascular injury, and diffusion MRI metrics (fractional anisotropy [FA] and mean diffusivity [MD]) for microstructural changes. Sentinel vQTLs were further examined using linear mixed models to pinpoint environmental exposures, such as air pollution, physical activity, and lifestyle factors, that mediate these associations.

**Result:**

The genome‐wide vQTL analysis identified five novel, genome‐wide significant loci associated with WMH burden (2q12.3, 3q27.3‐q28, 5p13.2, 10p11.22, and 17p11.2) along with four suggestive loci for HV and FA. Notably, associations at 2q12.3 (*ST6GAL2*), 10p11.22 (*ZEB1*), and 17p11.2 (*EPN2*) with WMH burden were significantly mediated by sedentary behavior and lifestyle factors (smoking, alcohol consumption). Functional insights suggest that *ZEB1* regulates gut microbiome species involved in inflammatory bowel diseases, while *ST6GAL2* has been implicated as an inflammatory biomarker associated with alcohol consumption. Additionally, differential gene expression analysis revealed significant downregulation of these risk loci in the spleen, with further enrichment observed in kidney cell‐type specific signatures.

**Conclusion:**

Our study identifies novel genome‐wide loci that interact with environmental factors and are associated with preclinical MRI markers of AD. These findings underscore the impact of modifiable lifestyle factors on genetic risk, offering potential avenues for preventive and therapeutic strategies. Ongoing efforts aim to replicate these findings in well‐characterized cohorts, including the Framingham Heart Study and the San Antonio Family Heart Study of predominantly Mexican‐Americans.

